# RhoB regulates cell migration through altered focal adhesion dynamics

**DOI:** 10.1098/rsob.120076

**Published:** 2012-05

**Authors:** Francisco M. Vega, Audrey Colomba, Nicolas Reymond, Mairian Thomas, Anne J. Ridley

**Affiliations:** Randall Division of Cell and Molecular Biophysics, King's College London, New Hunt's House, Guy's Campus, London SE1 1UL, UK

**Keywords:** Rho guanosine triphosphate, cytoskeleton, RhoB, focal adhesions, integrins, Rac1

## Abstract

The Rho GTPase RhoB has been shown to affect cell migration, but how it does this is not clear. Here we show that cells depleted of RhoB by RNAi are rounded and have defects in Rac-mediated spreading and lamellipodium extension, although they have active membrane ruffling around the periphery. Depletion of the exchange factor GEF-H1 induces a similar phenotype. RhoB-depleted cells migrate faster, but less persistently in a chemotactic gradient, and frequently round up during migration. RhoB-depleted cells have similar numbers of focal adhesions to control cells during spreading and migration, but show more diffuse and patchy contact with the substratum. They have lower levels of surface β1 integrin, and β1 integrin activity is reduced in actin-rich protrusions. We propose that RhoB contributes to directional cell migration by regulating β1 integrin surface levels and activity, thereby stabilizing lamellipodial protrusions.

## 2. Introduction

Rho GTPases are a family of signalling mediators implicated in regulating many processes, including cytoskeletal dynamics and motility, cell division, and transcriptional regulation. They contribute to various physiological functions both in normal and pathological situations, including wound healing, inflammation and cancer progression [1]. Most Rho family GTPases are active when bound to GTP and inactive when bound to GDP. They are activated by guanine nucleotide exchange factors (GEFs) and inactivated by GTPase-activating proteins. Active Rho GTPases bind to downstream effectors to induce cellular responses.

RhoB is most closely related to RhoA and RhoC, but has distinct biochemical and biological properties. RhoA, RhoB and RhoC are all post-translationally modified at their C-terminus by prenylation. RhoA and RhoC are only geranylgeranylated, whereas RhoB can be either geranylgeranylated or farnesylated. In addition, only RhoB is modified by palmitoylation [2,3]. RhoA and RhoC are localized mostly in the cytoplasm because they interact with RhoGDI, which extracts them from membranes by binding to the geranylgeranyl isoprenoid. By contrast, RhoB does not bind to RhoGDI, and is localized on the plasma membrane and/or on endosomes [4].

RhoB is known to regulate trafficking of growth factor tyrosine kinase receptors through endosomes, including epidermal growth factor (EGF) receptor and vascular endothelial growth factor (VEGF) receptor. It also affects trafficking of the non-receptor tyrosine kinase Src to the plasma membrane [5,6]. In RhoB knockout macrophages, integrin levels on the cell surface are reduced [7].

Previous results from our laboratory showed that RhoA and RhoC depletion induces distinct morphologies that result in different effects on cancer cell migration and invasion [8]. Here, we investigate the function of RhoB in regulating cell morphology and migration in cancer cells. We find that RhoB depletion inhibits cell spreading and stable lamellipodium extension, and promotes migration, but impairs persistence and directionality. Adhesion is not affected, but surface integrin β1 levels and its activity in protrusions are reduced, affecting focal adhesion contacts with the substratum during spreading or migration.

## 3. Results and discussion

### 3.1. RhoB depletion reduces cell spreading

**Figure 1.**
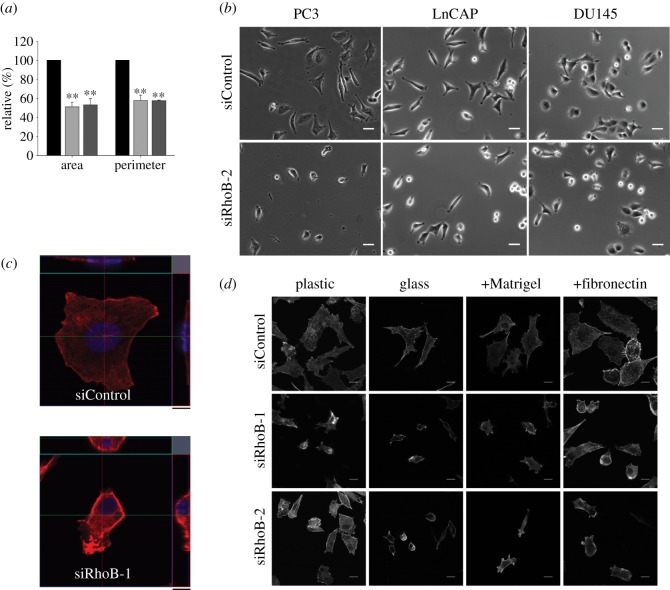
RhoB affects cell morphology. (*a*) Quantification of cell area and cell perimeter of siRNA-transfected PC3 cells (mean ± s.d. from four different experiments and >200 cells). Black bars denote siControl; light grey bars denote siRhoB-1; dark grey bars denote siRhoB-2. Values are shown relative to siControl (**p < 0.01). (*b*) Phase-contrast images of RhoB-depleted or control cells from the indicated cell lines. Scale bars, 40 µm. (*c*) Orthogonal view of a representative siControl or siRhoB-treated cell. Scale bars, *z* = 9.5 µm. (*d*) F-actin distribution in siRNA-transfected PC3 cells plated on uncoated plastic or glass, or plastic coated with Matrigel or fibronectin. Scale bars, 20 µm.

Using specific siRNAs, we found that RhoB-depleted PC3 cells had a reduced spread area and perimeter (figure 1*a*,*b*). They were also generally less flattened on the substrate (figure 1*c*). Similarly, RhoB depletion reduced spreading in LnCAP and DU145 prostate cancer cells (figure 1*b*), MDA-MB-231 breast cancer cells and primary endothelial cells (not shown); thus, the RhoB phenotype is not specific to a single cell type. RhoB depletion did not alter cell diameter in suspension, indicating that the lower spread area was not due to reduced size (see electronic supplementary material, figure S1*a*). The effect of RhoB on spreading did not depend on the substrate: RhoB-depleted cells were less spread on uncoated plastic or glass, or plastic coated with the extracellular matrix protein fibronectin or Matrigel (figure 1*d*). These results point to a central role of RhoB in the regulation of cell shape.

**Figure 2.**
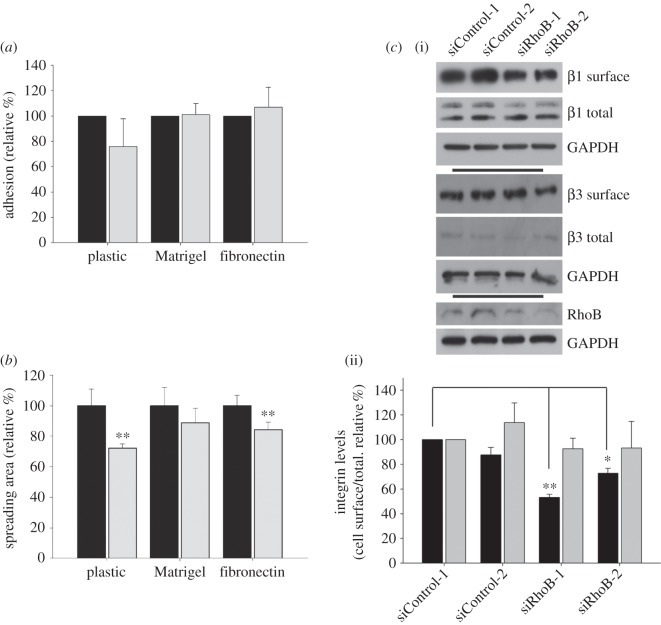
RhoB knockdown impairs spreading. (*a*) PC3 cell adhesion after 20 min and (*b*) spread area after 30 min on uncoated plastic, or plastic coated with Matrigel or fibronectin. Black bars denote siControl, grey bars denote siRhoB. Graphs show mean ± s.e.m. (*n* = 5 with >100 cells per condition; **p < 0.01). (*c*) Analysis of β1 and β3 integrin cell surface expression in PC3 cells. Surface proteins were biotinylated, pulled down with streptavidin beads and then analysed by immunoblotting with β1 (black bars) and β3 (grey bars) integrin antibodies and GADPH antibodies as a loading control. (i) Representative Western blot; (ii) mean levels ± s.e.m. of surface integrin levels relative to total and expressed as percentage of siControl-1 levels. *n* = 6. *p < 0.05, **p < 0.01.

The reduced spread area of RhoB-depleted cells could reflect decreased adhesion. RhoB depletion did not alter cell adhesion to uncoated plastic, Matrigel or fibronectin (figure 2*a*), whereas there was already a significant decrease in the spread area 30 min after seeding on plastic or fibronectin (figure 2*b*). Differences on Matrigel were not significant at 30 min, although reduced spreading was clearly observed at later points (see electronic supplementary material, figure S1*b*).

Macrophages derived from RhoB knockout mice have lower surface levels of β2 and β3 integrins, which could account for their reduced adhesion [7]. RhoB depletion reduced total surface levels of β1, but not β3 integrin (figure 2*c*), which could be responsible for the decreased spreading. Indeed, β1 integrin-null fibroblasts have reduced spread area, although they are able to adhere normally to fibronectin [9].

Together, these results indicate that RhoB-depleted PC3 cells are able to adhere normally to Matrigel or fibronectin, but fail to spread properly.

### 3.2. RhoB is essential for cell spreading downstream of Rac1

**Figure 3.**
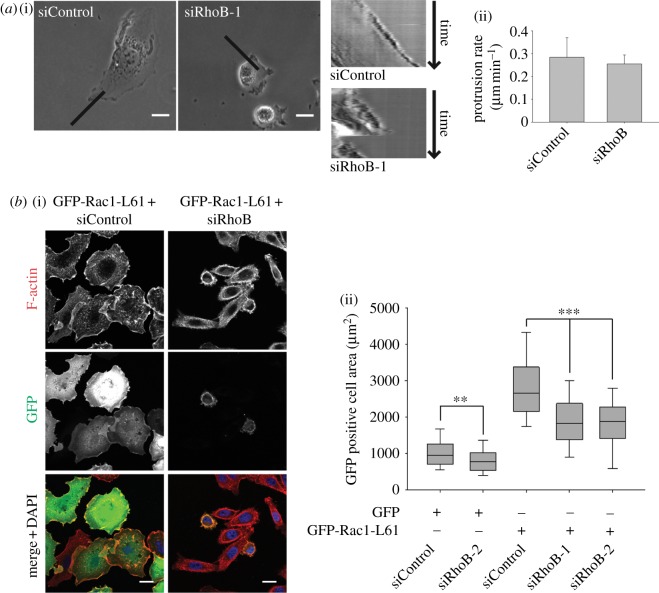
RhoB is required for Rac1-driven lamellipodial protrusion. (*a*) (i) Kymographs of representative lamellipodial regions of migrating siRNA-transfected PC3 cells from the electronic supplementary material, movie S1. Black bars denote kymograph region. Scale bars, 20 µm. (ii) Lamellipodial protrusion rate measured from five to seven cells per condition, mean ± s.d. (*b*) (i) Representative images and (ii) quantification of GFP-positive PC3 cells after transfection with GFP-Rac1-L61 or control GFP and the indicated siRNAs. Scale bars, 20 µm. *n* > 300 cells per condition from three independent experiments; boxes of box and whisker plots show median, 25th and 75th percentile; whiskers show 95th percentile; **p < 0.01, ***p < 0.001.

Analysis of time-lapse movies indicated that RhoB-depleted PC3 cells had a defect in spreading and lamellipodium stability, although dynamic membrane ruffles were frequently observed (see figure 3*a* and electronic supplementary material, movie S1). Multiple short protrusions were extended with intensive ruffling, but did not develop into a proper extended lamellipodium or a persistent leading edge. The speed of protrusion extension was similar between RhoB-depleted and control cells, but these protrusions were less persistent (figure 3*a*). The behaviour of the rear of migrating cells also appeared different: when a cell changed direction, the previous protrusion was now behind the cell and was then suddenly retracted, such that the rear and nucleus jumped forward. Altered adhesion, at the rear, can contribute to lamellipodium stabilization and polarization [10].

Rac1 is required for cell spreading and lamellipodium extension in many cell types, including PC3 cells [8]. Expression of constitutively active Rac1 induces cell spreading through extension of a broad lamellipodium around most of the cell periphery [11]. RhoB depletion reduced the spreading of both GFP-expressing control cells and cells expressing constitutively active GFP-Rac1-L61-expressing cells, showing that RhoB interfered with the formation of Rac1-induced lamellipodia (figure 3*b*). We showed previously that RhoB knockdown does not affect Rac1 activity in PC3 cells [8]. Moreover, RhoB depletion did not interfere with Rac1 signalling to its downstream target p21-activated kinase (PAK), as measured by steady-state levels of PAK phosphorylation (see electronic supplementary material, figure S1*c*). PAK is known to affect cell adhesion and spreading [12]. These results suggest that RhoB affects a basic pathway required for Rac-driven lamellipodium extension/stability rather than directly affecting Rac signalling.

### 3.3. RhoB-depleted cells have impaired directional migration

**Figure 4.**
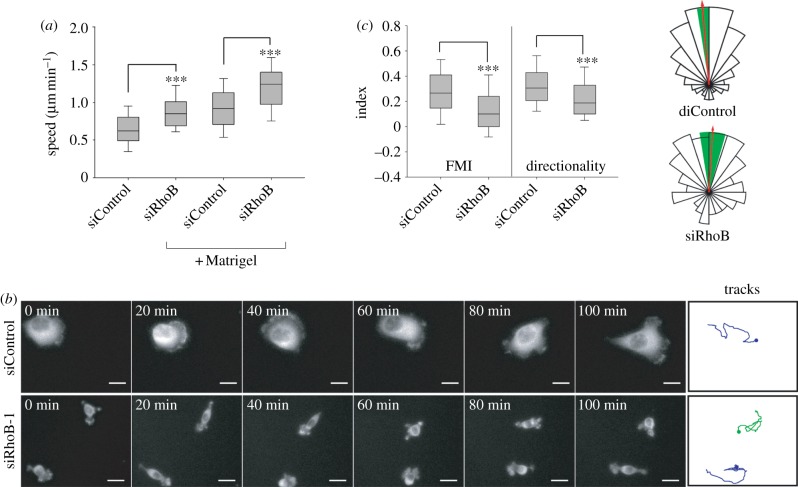
RhoB affects cell migration speed and directionality. (*a*) Migration speed of siRNA-transfected PC3 cells on tissue culture-treated plastic. *n* > 50 cells per condition from three different experiments. Results with two different siRNAs for RhoB and two siControl oligos are pooled; cells were tracked for 12–14 h, 58–60 h after transfection. (*b*) Montage from the electronic supplementary material, movie S3 showing representative GFP-actin-expressing PC3 cells. Tracks panels (right) show movement of the shown cells over time. Scale bars, 20 µm. (*c*) Forward migration index (FMI) and directionality of siRNA-transfected PC3 cells in a chemotaxis assay. *n* > 200 cells per condition from four different experiments using two different siRNAs for RhoB and two siControl oligos. Boxes of box and whisker plots show median, 25th and 75th percentile; whiskers show 95th percentile; ***p < 0.001. Rose plots show the proportion of cells with a migration direction lying within each 20° interval. Red arrow represents mean direction of cell migration; green segment, 95% CI.

The dramatic change in morphology and lamellipodial dynamics induced by RhoB knockdown suggested it would affect cell migration. RhoB-depleted PC3 cells showed a significant increase in migration speed both on uncoated and Matrigel-coated surfaces (see figure 4*a*; electronic supplementary material, movie S2). They also moved less persistently. This contrasts with our observations that RhoA or RhoC depletion reduces migration speed under similar conditions [8]. Cell tracking indicated that RhoB-depleted cells could extend a single small protrusion, polarize and migrate in one direction for a short time, but often went into a ‘tumbling’ phase when they extended multiple small protrusions in different directions, and then chose a different one to form a new leading edge (see figure 4*b*; electronic supplementary material, movies S3). By contrast, control cells had much broader and more persistent lamellipodia. As a result, RhoB-depleted cells moved faster, but with frequent turns. Similar migratory behaviour was observed in randomly migrating LnCAP or single DU145 cells (not shown).

To determine whether the frequent turning of RhoB-depleted cells affected directed migration, we studied their migration in a chemotactic gradient of serum. RhoB-depleted cells migrated towards the chemoattractant, but they were significantly less persistent than control cells (*p* < 0.0001), and had a reduced forward migration index and directionality following the gradient (figure 4*c*). The ability to follow the gradient was not affected according to the Rayleigh test for clustered direction vectors (CI: 99%; *p* < 0.0001), indicating that there was no defect in gradient sensing. Consistent with their overall reduced spread area (figure 1), RhoB-depleted cells frequently rounded up during migration, and in this state they showed little net movement (see electronic supplementary material, movie S4). They subsequently extended new protrusions, frequently not in the direction of the gradient, and moved again. By contrast, control cells tended to keep extending protrusions in the direction of the gradient. This behaviour could explain their reduced directional migration and persistence.

### 3.4. RhoB and GEF-H1 affect cell morphology but not microtubule dynamics

**Figure 5.**
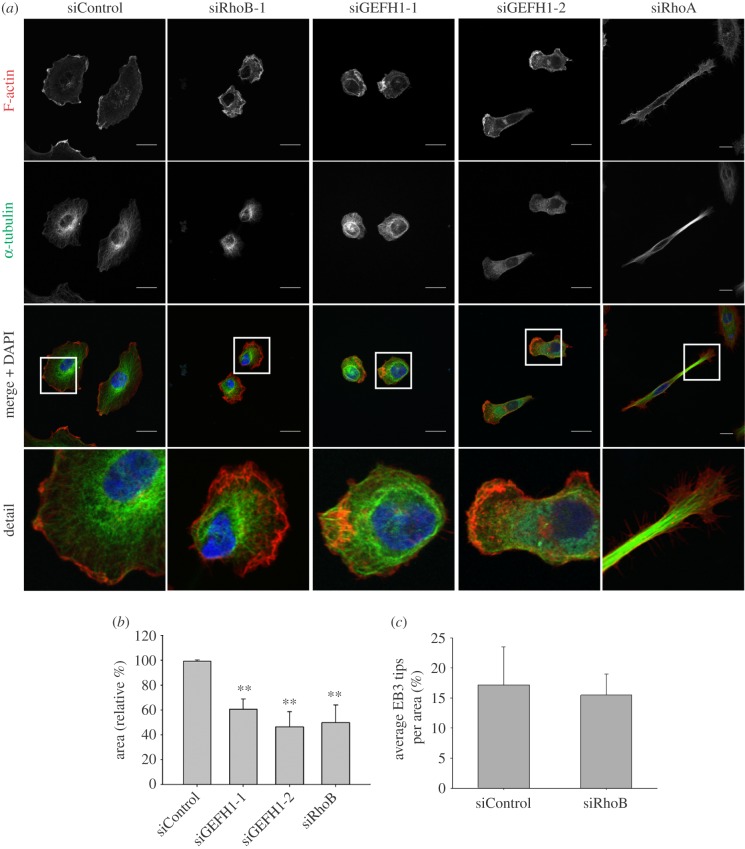
RhoB and GEF-H1 affect cell morphology, but not microtubule dynamics. (*a*) Confocal images showing F-actin and α-tubulin distribution in PC3 cells transfected with siRNAs targeting GEF-H1, RhoA or control siRNA. Scale bars, 20 µm. (*b*) Graph shows quantification of cell area (mean ± s.e.m.) relative to siControl from two different experiments with >40 cells per condition; **p < 0.01. (*c*) Quantification of microtubule tips per unit area from movies of EB3-GFP-expressing RhoB-depleted or control cells. Graph shows mean ± s.e.m. from 10 cells in two different experiments.

**Figure 6.**
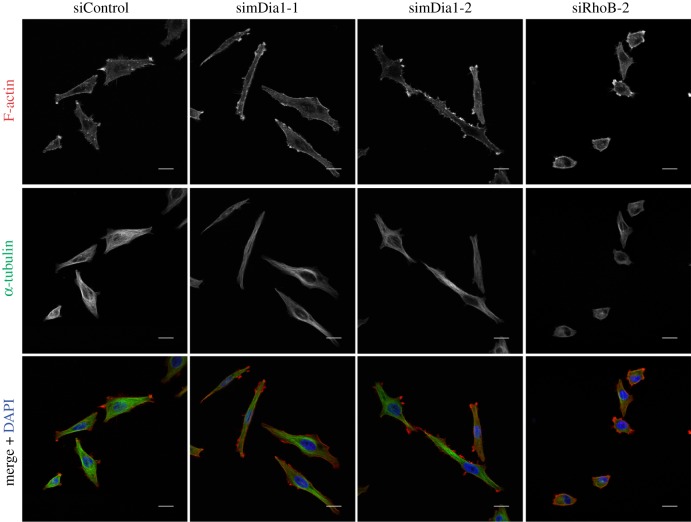
Effects of mDia1 depletion on PC3 cells. Cells were transfected with two different siRNAs targeting mDia1, RhoB or control siRNA. Confocal images show F-actin and α-tubulin distribution. Scale bars, 20 µm.

Integrin-mediated adhesion and lamellipodial dynamics are regulated by microtubule dynamics in addition to actin polymerization [13,14]. We therefore investigated whether the effects of RhoB were due to changes in microtubules. RhoB-depleted cells did not have a different microtubule organization, although microtubules appeared condensed around the periphery in some cases owing to the reduced spreading (figure 5*a*). Results from a siRNA screen indicated that knockdown of the RhoGEF GEF-H1 (officially known as ARHGEF2) could affect cell morphology (FM Vega 2011, unpublished data). GEF-H1 is best known as an activator of RhoA [15,16], but can also act as a GEF for RhoB [17]. GEF-H1 depletion in PC3 cells reduced cell spread area, similar to RhoB knockdown and clearly different to the elongated phenotype observed in RhoA-depleted cells (figure 5*a* [8]). GEF-H1 binds directly to microtubules, and GEF-H1 knockdown in HeLa cells was reported to increase the number of microtubules reaching the cell periphery [16]. We therefore analysed microtubule dynamics in PC3 cells transfected with EB3-GFP, which localizes to microtubule tips. From the analysis of movies, we did not observe any change in the number of EB3-GFP3-positive microtubule tips at the cell edge (see figure 5*c*; electronic supplementary material, figure S2*a*). In addition, neither RhoB nor GEF-H1 depletion affected the levels of stabilized acetylated tubulin (see electronic supplementary material, figure S1*d*,*e*). RhoB can interact with mDia formins, which are known to regulate both actin polymerization and microtubule dynamics [18]. However, mDia1-depleted PC3 cells had an elongated morphology (figure 6), consistent with our previous results [19]. This did not resemble the reduced spreading phenotype of RhoB depletion. The RhoB knockdown phenotype was also very different to that of PC3 cells depleted of the Rho-kinases ROCK1 or ROCK2 [8]. It is therefore unlikely that either mDia1 or ROCKs mediate RhoB-dependent morphological changes in these cells.

These results show that both RhoB and GEF-H1 regulate cell spreading, but this does not appear to be due to altered microtubule stability or tip dynamics.

### 3.5. RhoB affects focal contact dynamics and β1 integrin activity

**Figure 7.**
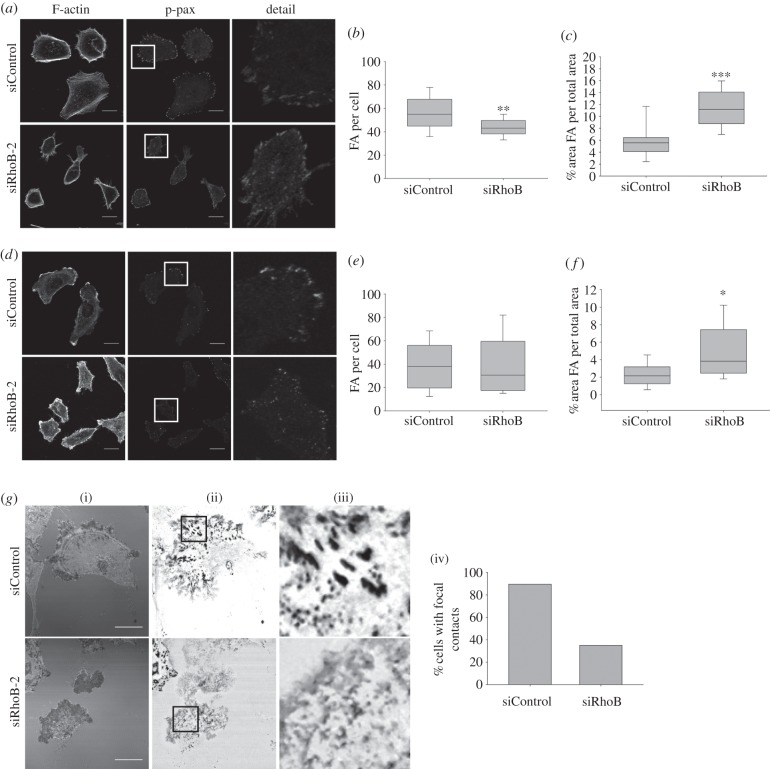
RhoB regulates focal adhesion dynamics. (*a*) Serum-starved PC3 cells stimulated with 10% FCS for 1 h to promote spreading were stained for F-actin and with antibodies to phospho-paxillin to show focal adhesions and focal complexes. Boxed regions (middle panels) are shown at higher magnification in the right panels. (*b*,*c*) Graphs show the number of phospho-paxillin-positive focal adhesions/focal complexes (FA) per cell or area occupied by FA relative to the total spread area. (*d*) PC3 cells under normal migratory conditions (1% FCS) were stained for F-actin and with antibodies to phospho-paxillin to show FA. Boxed regions (middle panels) are shown at higher magnification in the right panels. (*e*,*f*) Number of phospho-paxillin-positive FA per cell or area occupied by FA relative to the total spread area in normal migratory conditions. Results in graphs are from at least 10 cells from two different experiments. Boxes of box and whisker plots show median, 25th and 75th percentile; whiskers show 95th percentile; ***p < 0.001, **p < 0.01, *p < 0.05. (*g*) (i) Representative interference reflection microscopy images of PC3 cells after siRNA transfection. (ii) Dynamics images show a thresholded maximal projection of the same cells over 70 frames (15 s frame^−1^). (iii) Detail: enlargement of boxed regions in (ii). (iv) Graph shows percentage of cells with stable focal contacts in interference reflection microscopy movies; *n* > 20 cells per condition. (*a*,*d*,*g*) Scale bars, 20 µm.

Because the level of total surface β1 integrin was reduced by RhoB depletion (figure 2*c*), integrin-mediated adhesions could be affected by RhoB. Cells were stained with antibodies to phospho-paxillin, which localizes to focal adhesions and smaller focal complexes [20]. Cells were analysed during both acute serum-induced spreading that stimulates focal complex and adhesion formation (collectively called FA here), and under the normal migratory conditions (1% foetal calf serum, FCS) used for other experiments (compare figure 7*a* and *d*). RhoB-depleted cells had a reduced spread area following acute serum-induced spreading, as observed for cells under normal conditions (see electronic supplementary material, figure S2*b*; figure 1*a*). Serum-stimulated RhoB-depleted cells had less FA/cell than control cells (figure 7*b*), suggesting a defect in the formation or increased turnover of new FA. In contrast, under the steady-state normal migratory conditions, the number of FA/cell was similar between the two populations (figure 7*e*). The area occupied by FA compared with the total spread area measured was increased in RhoB knockdown cells in both conditions, because the spread area of these cells is lower than control cells (figure 7*c*,*f*).

To observe the dynamics of adhesions, sites of cell contact with the substratum were monitored by time-lapse interference reflection microscopy (IRM) [21]. Most migrating PC3 cells had stable sites of focal contact with the substratum at the leading edge and on their ventral surface (figure 7*g*). By contrast, RhoB knockdown cells often had more diffuse and short-lived areas of contact with the substratum, which turned over more rapidly (see figure 7*g*; electronic supplementary material, movie S5). This correlates with the smaller and more widely distributed FA (figure 7*d*).

**Figure 8.**
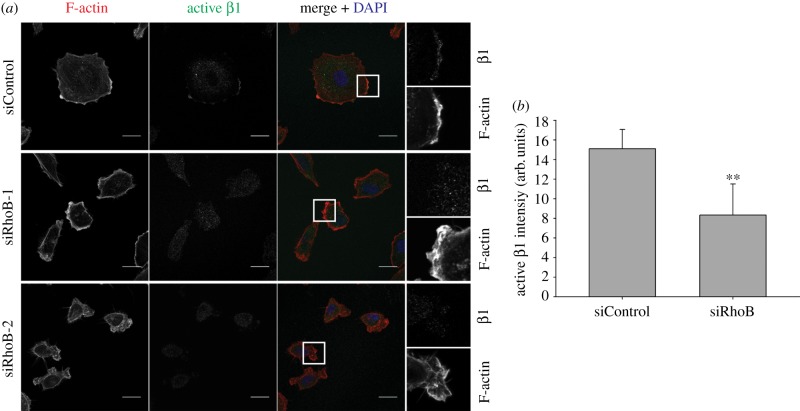
RhoB affects β1 integrin activity in protrusions. (*a*) Confocal images showing active β1 integrin staining and F-actin. Boxed regions (merge panels) are shown at higher magnification in the right panels. Scale bars, 20 µm. (*b*) Quantification of active β1 integrin intensity in protrusions. Arb. units denotes arbitrary units. *n* = 18 cells from two different experiments; **p < 0.01.

Because β1 integrin surface expression was regulated by RhoB, we explored whether β1 integrin activity at the plasma membrane could be affected. Active β1 integrin can be detected with conformation-sensitive antibodies, and localizes to lamellipodia [9]. Despite the active ruffling and the presence of small actin-rich protrusions in RhoB-depleted cells, a significant reduction in active β1 integrin was observed in these areas (figure 8), suggesting that RhoB could regulate cell spreading by promoting β1 integrin activation.

## 4. Conclusions

We hypothesize that RhoB normally regulates cancer cell migration by increasing the stability of integrin-mediated adhesions and integrin activity, thereby promoting lamellipodial protrusion and migratory polarity. RhoB has been implicated in the recycling and delivery of receptors and signalling proteins to the plasma membrane via its action on endosomal trafficking [22–24], and thus it is possible that some component required for integrin activation and focal contact stability is delivered to the leading edge by RhoB. We monitored Src because it mediates integrin signalling and its trafficking is regulated by RhoB [23,25]. Src activity was unchanged in RhoB-depleted PC3 cells (see electronic supplementary material, figure S1*b*) and Src localized normally in peripheral membrane ruffles (data not shown), thus it is unlikely that Src plays a role in the RhoB-mediated effect on focal contacts. Focal adhesion positioning, on the other hand, has been described to control directional cell motility [26].

RhoB expression is reduced in several tumour types compared with non-cancer tissues and can contribute to tumour growth by regulating apoptosis [27]. Its tumour suppressor activity could also reflect a role in inhibiting cancer cell migration. Interestingly, RhoB expression is frequently induced by stress stimuli (including DNA damage) and by growth factors [27], and thus it might acutely modulate cell shape and migration in response to these treatments.

## 5. Material and methods

### 5.1. Cell lines and reagents

PC3, DU145 and LnCAP prostate cancer cell lines were grown in RPMI containing 25 mM Hepes, 2 mM glutamine, 10 per cent FCS, 100 μg ml^−1^ streptomycin and 100 units ml^−1^ penicillin. The following antibodies were used: RhoB (sc-180) and rabbit polyclonal ERK (K-23, Santa Cruz), α-tubulin (DM1A clone, Sigma), rabbit polyclonals Pak1, p-Pak1(Ser199/204), p-Src(Tyr416), p-Paxilin(Tyr118) and GEF-H1 (Cell Signaling), mouse monoclonal Src and acetylated-tubulin (Upstate), mouse anti-human active β1 integrin 9G10 (AbCam), mouse anti-human β1 (clone 4B7R, AbCam) and β3-Integrin (clone 25E11, Millipore). Secondary horseradish peroxidase (HRP)-labelled antibodies were from Amersham.

### 5.2. Transfections and immunoblotting

siRNAs were from Dharmacon (ThermoScientific): siRhoB-1 (CAUCCAAGCCUACGACUAC), siRhoB-2 (GCAUCCAAGCCUACGACUA), siRhoB-3 (GCAUCCAAGCCUACGACUA), siRhoA (AUGGAAAGCAGGUAGAGUU), GEF-H1-1 (CAACAUUGCUGGACAUUUC), GEF-H1-2 (GAAUUAAGAUGGAGUUGCA), simDia1-1 (GAAGUGAACUGAUGCGUUU), simDia1-2 (GAAGAGAGAGCAACUCAUA) and ON-TARGET *plus* non-targeting siControls (D-001810-01, D-001810-02). All siRNAs were initially tested for protein knockdown. Cell transfection, lysate preparation and immunoblotting were performed as described previously [8]. Cells were analysed 72 h after siRNA transfection.

Constructs encoding GFP, GFP-Rac1-L61, GFP-β-actin and EB3-GFP were transfected into PC3 cells using JETPRIME reagent following the manufacturer's instructions (PolyPlus transfections), 48 h after siRNA transfection. Cells were analysed 20 h after DNA transfection.

### 5.3. Time-lapse microscopy

For bright field time-lapse microscopy, a fully motorized, multi-field Nikon TE2000 microscope was used. Random migration and chemotaxis experiments were carried out as described [8]. For chemotaxis assays, collagen IV pre-coated microslides (Ibidi) were used according to the manufacturer's instructions. Chemotaxis and persistence were analysed using the ImageJ Manual Tracking plugin and a customized Mathematica chemotaxis notebook (M6) written by Graham Dunn (parameters for motility analysis: dt = 8, TR = 4) [28]. The Rayleigh test for unimodal clustering of directions [29] was applied to the chemotaxis tracking data, and *p* < 0.01 was chosen as the criterion for rejecting the null hypothesis of random directionality. Kymographs were generated, and protrusion rate calculated using Metamorph software.

### 5.4. Confocal microscopy and cell shape analysis

Immunofluorescence staining was carried out as previously described [8]. Alexa Fluor Phalloidin (wavelengths 480 nm, 543 nm or 633 nm, Molecular Probes) was used for F-actin visualization, DAPI for nuclear staining and FITC-labelled α-tubulin antibody (DM1A clone) for microtubule staining. Confocal images were acquired with a Zeiss LSM510 inverted confocal microscope. Morphology analysis was carried out using Metamorph or Cell Profiler software [30] from F-actin-stained fluorescence images. Active β1 integrin levels in F-actin-rich protrusions were quantified by measuring pixel intensity following 9G10 active β1 integrin antibody staining in regions defined by the presence of strong F-actin staining at the cell periphery.

### 5.5. Cell adhesion and spreading

For cell adhesion quantification, siRNA-transfected cells were detached with versene, labelled with 2 μM carboxyfluorescein diacetate succinimidyl ester (Invitrogen) and seeded on uncoated culture plates or plates previously coated with 10 μg ml^−1^ fibronectin (Sigma-Aldrich) or 100 μg ml^−1^ Matrigel (BD Biosciences). After 20 min, culture plates were washed with phosphate-buffered saline (PBS), and the number of adherent cells were quantified with a Fusion α-FP plate reader (PerkinElmer) using an excitation of 485 nm and an emission filter of 525/35 nm. Alternatively, cells were fixed with 4 per cent paraformaldehyde and counted under the microscope to corroborate the plate reader results. For cell spread area measurement, cells were allowed to spread on the indicated substrates for different time points and fixed with paraformaldehyde without washing. Cells were then stained for F-actin, and area and perimeter quantified with Cell Profiler software.

### 5.6. Surface biotinylation assay

After 72 h of siRNA transfection, cells were washed three times in cold PBS and surface-labelled at 4°C with 0.3 mg ml^−1^ NHS-LC-biotin (Thermo Scientific) in PBS for 30 min. Labelled cells were washed four times in cold PBS, and excess of biotin was removed by incubation with RPMI 10 per cent FCS for 20 min at 4°C. After three PBS washes, cells were lysed in 50 mM Tris pH 8, 150 mM NaCl, 5 mM EDTA, 1 per cent NP-40, 25 mM NaF, 2 mM Na_3_VO_4_, phosphatase inhibitor cocktail (Calbiochem) and protease inhibitor cocktail (Roche). Lysates were sonicated, clarified by centrifugation at 16 000*g* for 10 min and incubated with streptavidin agarose beads (Thermo Scientific) for 2 h at 4°C. Beads were washed four times with lysis buffer, and pulled down β1 and β3 integrins were analysed by SDS–PAGE and Western blotting.

### 5.7. Interference reflection microscopy analysis

IRM microscopy was carried out on a Zeiss LSM510 inverted confocal microscope with a 63× objective and 488 nm laser. siRNA-transfected PC3 cells were seeded on glass-bottom dishes (MatTek). IRM images were acquired at 15 seconds per frame for 1 h. To analyse focal contact dynamics, images were processed in ImageJ software following a simplified version of the process described previously [21]. Cells were scored as having focal contacts if over time they developed dense, discrete contact points with the substrate when migrating, which could be observed as dense dark structures in the images resulting from the analysis.

### 5.8. Analysis of microtubule dynamics and quantification of focal adhesions/complexes

Analysis of microtubule tip dynamics was carried out essentially as described previously [16] using movies from PC3 cells over-expressing EB3-GFP and treated with the indicated siRNAs. Cell Profiler was used for segmentation and quantification of number of EB3-GFP-labelled tips around the cell periphery. A similar pipeline was used to quantify focal adhesions/complexes from images of cells stained with anti-phospho-paxillin antibody and fluorophore-conjugated phalloidin to show actin filaments. Cells were either serum starved and stimulated to spread with 10 per cent FCS for 1 h before fixing (acute serum-induced spreading) or grown in medium containing 1 per cent FCS on glass coverslips (normal migrating condition).

### 5.9. Statistical analysis

For statistical significance analysis either an unpaired two-tailed *t*-test (for comparing two conditions) or a one-way ANOVA with Dunn's multiple comparison post test (for comparing multiple conditions) was used. All significances indicated are compared with siControl condition unless stated. At least two different siRNAs for each gene were analysed in every assay and results were pooled in some graphs.

## Supplementary Material

Supplementary Figures
