# Supplementary material for: RhoB regulates cell migration through altered focal adhesion dynamics
Source: Open Biol. 2012 May;2(5):120076. doi: 10.1098/rsob.120076 (PMC3376739; doi:10.1098/rsob.120076)
Supplement: Supplementary Figures [file rsob120076-s1.pdf]

Figure S1

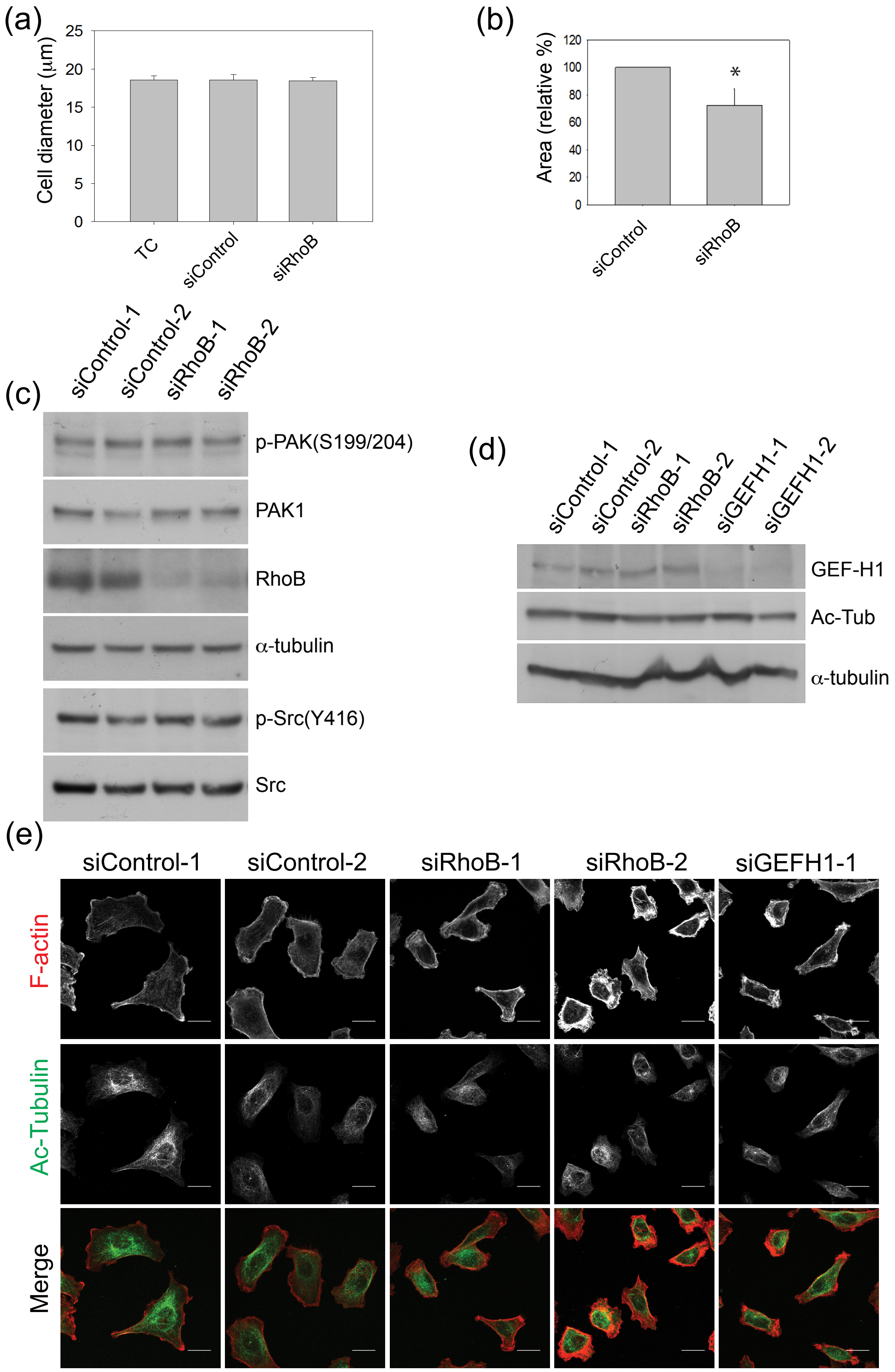

Figure S1. RhoB or GEF-H1 depletion does not affect PAK, Src or microtubules. PC3 cells transfected with siRNAs to RhoB, GEF-H1 or control siRNAs were analysed 72 h after transfection. (a) Quantification of cell diameter in transfected PC3 cells in suspension. TC: transfected control. (b) Quantification of cell area in siRNA-transfected PC3 cells on Matrigel-coated plastic 48 h after adhesion. (c) Immunoblot showing the levels of the indicated proteins or phospho-proteins. (d) Immunoblot showing acetylated tubulin (Ac-Tub) and total tubulin levels. (e) Immunofluorescence images showing acetylated tubulin (Ac-tubulin) and F-actin distribution. Bars, 20 µm.

Figure S2

(a)

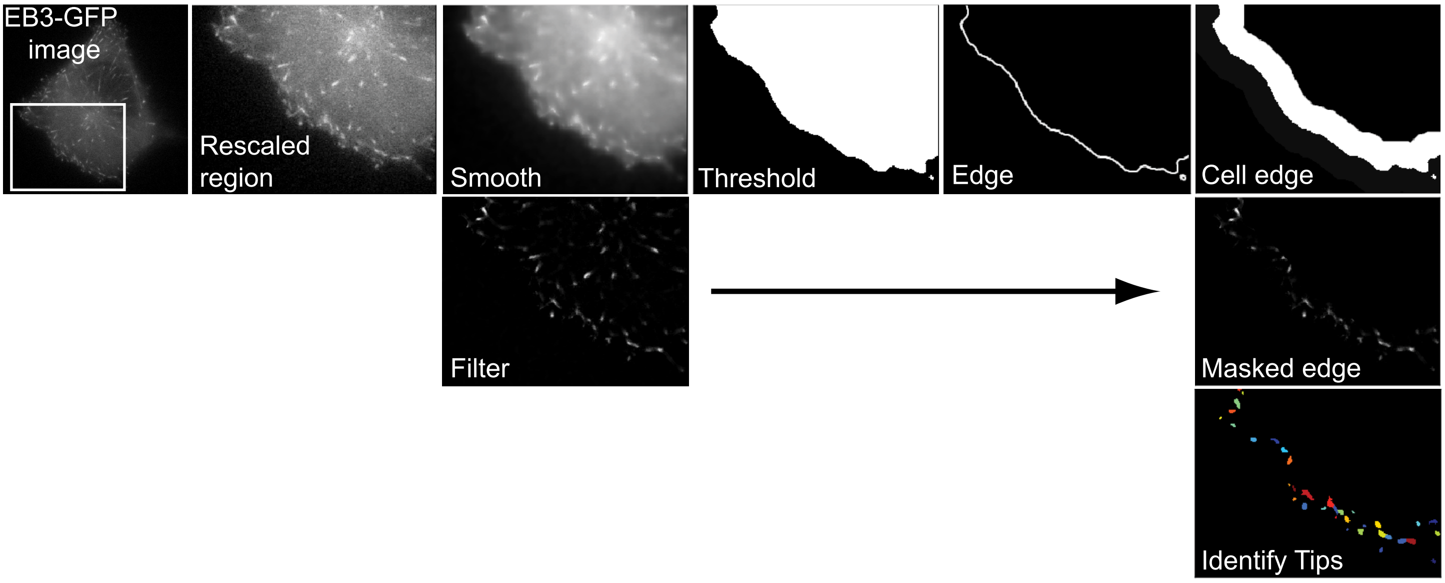

(b)

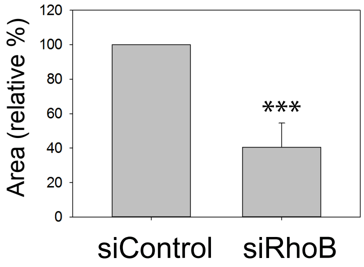

Figure S2. Quantification of microtubule tips and effects of RhoB on cell spread area. (a) Pipeline showing method for quantifying EB3-positive microtubule tips at the cell periphery from fluorescence movies of PC3 cells expressing EB3-GFP. (b) Graph showing spread area of serum-starved PC3 cells stimulated with 10% FCS for 1 h to promote spreading. Results in graphs are from at least 20 cells from two different experiments. Boxes of box and whisker plots show median, 25th and 75th percentile; whiskers show 95th percentile; ***$p<0.001$.
